# Increased efficiency of evolved group I intron spliceozymes by decreased side product formation

**DOI:** 10.1261/rna.051888.115

**Published:** 2015-08

**Authors:** Zhaleh N. Amini, Ulrich F. Müller

**Affiliations:** Department of Chemistry and Biochemistry, University of California, San Diego, La Jolla, California 92093-0356, USA

**Keywords:** ribozyme, *trans*-splicing, evolution in cells, side products

## Abstract

The group I intron ribozyme from *Tetrahymena* was recently reengineered into a *trans*-splicing variant that is able to remove 100-nt introns from pre-mRNA, analogous to the spliceosome. These spliceozymes were improved in this study by 10 rounds of evolution in *Escherichia coli* cells. One clone with increased activity in *E. coli* cells was analyzed in detail. Three of its 10 necessary mutations extended the substrate binding duplexes, which led to increased product formation and reduced cleavage at the 5′-splice site. One mutation in the conserved core of the spliceozyme led to a further reduction of cleavage at the 5′-splice site but an increase in cleavage side products at the 3′-splice site. The latter was partially reduced by six additional mutations. Together, the mutations increased product formation while reducing activity at the 5′-splice site and increasing activity at the 3′-splice site. These results show the adaptation of a ribozyme that evolved in nature for *cis*-splicing to *trans*-splicing, and they highlight the interdependent function of nucleotides within group I intron ribozymes. Implications for the possible use of spliceozymes as tools in research and therapy, and as a model for the evolution of the spliceosome, are discussed.

## INTRODUCTION

Group I introns are catalytic RNAs (ribozymes) that are encoded as intervening sequences in pre-mRNAs. In contrast to most introns, group I introns do not require the spliceosome for their removal from pre-mRNA. Instead, they fold into structures that catalyze their own excision from the pre-mRNA and the joining of the flanking exons (Kruger et al. 1982). Group I introns from different species have a conserved core structure that includes the catalytic site, but they differ in the peripheral regions (Guo et al. 2004; Golden et al. 2005; Stahley and Strobel 2005). The most detailed biochemical characterization is available for the group I intron from *Tetrahymena* (Cech 1990; Michel and Westhof 1990; Stahley and Strobel 2006). This ribozyme is robust with regard to sequence modifications in its peripheral domains, and it is active in vitro, in bacteria, and eukaryotes including yeast, mouse, and human cells.

**FIGURE 1.**
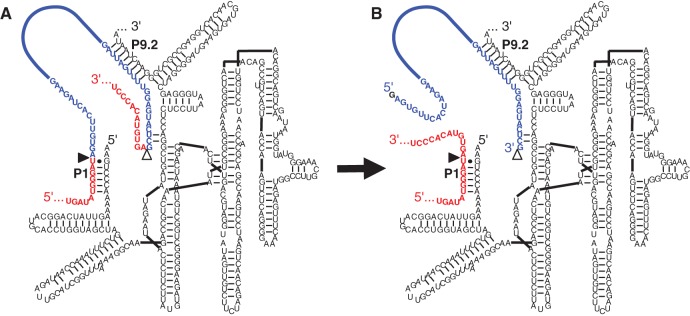
Secondary structure of spliceozyme used as parent in the evolution. The spliceozyme (black) base pairs with the *CAT* pre-mRNA (red/blue) by forming duplexes P1 and P9.2. (*A*) Before the splicing reaction, the pre-mRNA contains the 5′-exon (red), a 100-nt intron (blue), and the 3′-exon (red). (*B*) After the splicing reaction, the 5′-exon and 3′-exon are joined to form *CAT* mRNA (red), and the intron (blue) is excised. The 5′-splice site is indicated by a filled triangle; the 3′-splice site is indicated by an open triangle. Note that the intron 5′ terminus is extended by an external guanosine during the reaction.

Variants of group I introns have been engineered to splice a substrate RNA in *trans*. Group I introns that splice in *trans* at their 5′-splice site are able to bind to a substrate RNA and replace the substrate's 3′ terminus with the ribozyme's own 3′-exon (Sullenger and Cech 1994). Similarly, *trans*-splicing at the 3′-splice site allows replacement of the substrate 5′ terminus with the ribozyme's 5′-exon (Alexander et al. 2005). *Trans*-splicing at both splice sites allows the removal of single nucleotides of a substrate RNA in cells, and up to 28 nt in vitro (Bell et al. 2002; Dotson et al. 2008). Using a different design principle for the substrate recognition at both splice sites, a *Tetrahymena* group I intron was recently shown to excise 100-nt-long introns from pre-mRNAs in vitro and in bacterial cells, efficiently enough to mediate antibiotic resistance (Amini et al. 2014). These ribozymes were termed “spliceozymes” because their action is analogous to that of the spliceosome (Fig. 1).

Spliceozymes may have the potential for applications as tools in therapy and in research. This system could be used to correct mis-splicing resulting from aberrant splicing diseases (Ward and Cooper 2010), to switch between splicing isoforms for the study of spliceosomal splicing, and as a model system to study the biochemical steps that likely occurred in the evolution of the spliceosome from a common ancestor with group II intron ribozymes (Sharp 1985; Cech 1986; Fica et al. 2013). For the use of spliceozymes as tools, however, several hurdles need to first be overcome. For use in therapy, efficient delivery methods need to be developed. There are current efforts to develop adeno-associated virus vectors (Ketzer et al. 2012), cationic peptides (Ewert et al. 2008), nanoparticles (Chen et al. 2012; Namiki 2013), and modified *Salmonella* strains (Yuhua et al. 2001; Bai et al. 2011) as delivery vehicles, yet improvements to minimize toxicity and immune responses and increase delivery to target tissues are still needed. Additionally, for the use as a tool in therapy or research, improvements are required to increase splicing efficiency in cells, and reduce off-target effects. This study aims to further develop spliceozymes for use in therapy and research by utilizing evolution to increase splicing efficiency in cells.

In addition to the use of spliceozymes as tools, this system is also able to serve as a model system to study the biochemical steps in the evolution of the spliceosome. While the spliceosome likely evolved from a common ancestor with group II introns (Fica et al. 2013), group I introns can be used as analogs to model comparable biochemical steps. The existing *trans*-splicing group I intron spliceozymes show that *cis*-splicing ribozymes can make the evolutionary step to *trans*-splicing ribozymes (Amini et al. 2014), analogous to perhaps the first step in the evolution of the spliceosome from a *cis*-splicing ancestor. Among the steps still required to obtain an analog of the spliceosome, the existing spliceozymes need (1) an increase in *trans*-splicing efficiency, (2) a reduction in side products and off-target effects, (3) the ability to recognize different splice sites, (4) the recruitment of proteins for structural and catalytic roles, and (5) fragmentation into multiple RNA–protein particles. This study shows that the first two steps can be addressed by an evolutionary system in cells.

Here we show the improvement of spliceozymes by directed evolution in *E. coli* cells. Over 10 rounds of evolution, spliceozymes were challenged to remove an intron from the pre-mRNA of chloramphenicol acetyltransferase (CAT) under increasingly stringent conditions. Analysis of one of the fittest spliceozymes, containing 10 mutations relative to the parent spliceozyme, showed increased *trans*-splicing efficiency and reduced side product formation. Three mutations extended the substrate recognition sequences, resulting in increased product formation and reduced side product formation. Additionally, a mutation in the conserved core of the spliceozyme (U271C) reduced activity of the 5′-splice site and increased activity at the 3′-splice site. A structural model is presented for a possible mechanism of this core mutation. A number of additional mutations were able to mitigate the increase in side product formation, which resulted from increased activity at the 3′ splice site. The implications for possible uses of spliceozymes as tools and as model systems for ribozyme evolution are discussed.

## RESULTS

**FIGURE 2.**
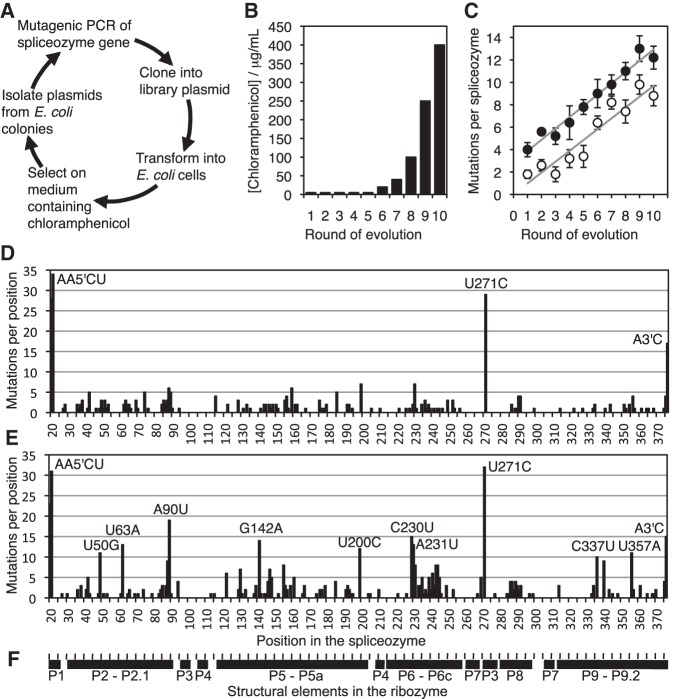
Evolution of spliceozymes in *E. coli* cells. (*A*) Schematic of the evolution procedure. (*B*) Selection pressure as a function of the rounds of evolution. The concentration of chloramphenicol was 5 μg/mL for the first five evolution rounds. Both lines of evolution were subjected to the same profile. (*C*) Average number of mutations per spliceozyme sequence over 10 rounds of evolution. Empty circles show the mutations in the evolutionary line starting from the wild-type sequence (W), and filled circles the mutations in the evolutionary line starting from a pre-evolved pool (P). Linear fits resulted in a slope of 0.97 for line W, and 1.01 for line P. The *y*-axis offset was 0.0 for line W, and 2.9 for line P. Error bars are the uncertainties of the mean from five sequences (rounds 1–9), or 10 sequences (round 10). (*D*) Positions of the mutations in the spliceozyme sequence for line W, summed over 10 rounds of evolution. The height of each column is the number of mutations per position within 55 sequences. Mutations that occurred at least 10 times are annotated with the mutation that occurred most frequently. Mutations at the spliceozyme 5′ terminus and 3′ terminus are labeled with 5′ and 3′, respectively. (*E*) As in *D* but for line P. (*F*) Secondary structure elements of the spliceozyme, aligned with the nucleotide positions in the graphs of *D* and *E*.

To improve the function of spliceozymes in cells, spliceozymes were subjected to directed evolution in *E. coli* cells (Fig. 2A) using an evolution system established in earlier studies (Olson and Muller 2012; Olson et al. 2014). As starting points for the evolution, two spliceozyme libraries were generated by mutagenic PCR, using as template either a single spliceozyme gene (Fig. 1; Amini et al. 2014) or a pre-evolved library that already contained genetic diversity which, in some circumstances, can increase the rate at which new ribozyme characteristics are accessed by evolution (Hayden et al. 2011). The spliceozyme libraries were cloned into the spliceozyme expression cassette of a library plasmid that also contained the expression cassette of the pre-mRNA of chloramphenicol acetyl transferase (CAT). After transformation into *E. coli* cells, the cells were plated on medium containing chloramphenicol. Only cells expressing functional spliceozymes, able to remove the intron from the *CAT* pre-mRNA, generated functional CAT enzyme, and formed colonies. The plasmids from these colonies were isolated to complete one round of evolution.

A low selection pressure was applied for the first five rounds of evolution, to allow for more efficient sampling of sequence space (Amini and Muller 2013). The selection pressure was successively increased over the last five cycles of evolution to isolate the most efficient ribozyme variants generated (Fig. 2B). Sequencing of five to 10 spliceozyme clones in each evolution round, for each of the two lines of evolution, showed that the number of mutations increased by approximately one mutation per spliceozyme per evolution round (Fig. 2C). Four mutations were highly enriched (Fig. 2D,E): Two mutations at the spliceozyme 5′ terminus (AA5′CU), one mutation in the core of the ribozyme (U271C), and one mutation at the ribozyme 3′ terminus (A3′C). Interestingly, enrichments of the U271C and 5′ terminus mutations were observed concurrently, both being enriched above 20% by round 4 or 5, while equivalent enrichment of the 3′ terminus mutation was not observed until round 8 (Supplemental Fig. S1). The enriched U271C mutation in the conserved core of the ribozyme was surprising; mutations in this region generally lead to severe functional deficiencies (Michel and Westhof 1990).

**FIGURE 3.**
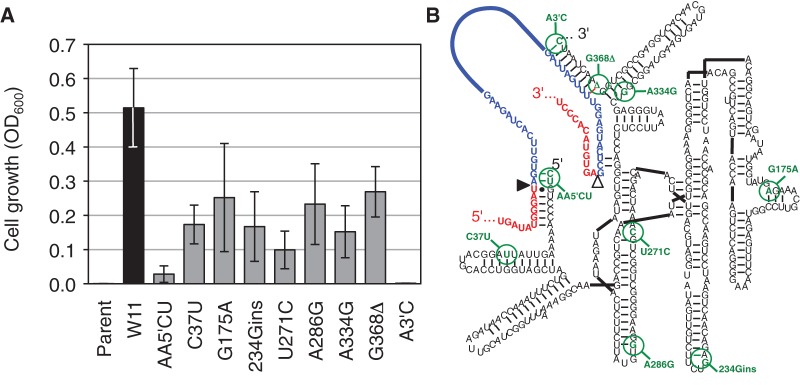
Identification of mutations necessary for full growth levels in the evolved spliceozyme W11. (*A*) Growth of *E. coli* cells expressing different spliceozyme variants on plates containing 100 μg/mL of chloramphenicol. In addition to the parent ribozyme and the evolved ribozyme W11, variants of W11 with reversions of indicated mutations were analyzed. The reverted mutations are given below the graph, with AA5′CU describing the mutation of two 5′-terminal nucleotides to CU, and A3′C describing the mutation of the 3′-terminal nucleotide to C. The OD_600_ was measured from growth on plates, and was normalized to the OD_600_ from plate cultures containing ampicillin (i.e., viable cells containing plasmid). Error bars are standard deviations from biological triplicates. (*B*) Secondary structure of the evolved spliceozyme W11, with evolved mutations indicated in green and with green circles. New base pairs created by the mutation are highlighted in green, and base pairs deleted by the mutation are highlighted in red.

To identify the most active sequences resulting from the evolution, the spliceozyme genes of 20 clones were recloned and tested for their ability to mediate bacterial growth on medium containing chloramphenicol (data not shown). Of the two clones mediating most efficient growth, one clone (termed W11) contained the central U271C mutation and was analyzed in more detail (Fig. 3). Clone W11 originated from the line of evolution that started with an individual sequence, and contained 10 mutations relative to the parent spliceozyme. To identify the mutations necessary for maximum growth, each mutation was individually reverted to the parent sequence and the resulting growth was measured. Surprisingly, all 10 mutations were necessary for full growth of *E. coli* cells. The strength of effects correlated with the enrichment during evolution: The strongest effects were mediated by the spliceozyme mutations at the 5′ and 3′ terminus (which extend the substrate recognition duplexes by 2 and 1 nt, respectively) followed by the mutation U271C. The six additional mutations were also required for full growth (C37U, G175A, 234Gins., A286G, A334G, and G368Δ). These six mutations were not specifically enriched during the evolution (Fig. 2D,E) or associated with other mutations (data not shown). To simplify the analysis of mutations, these six mutations (termed “helper mutations”) were grouped together and studied in combination.

**FIGURE 4.**
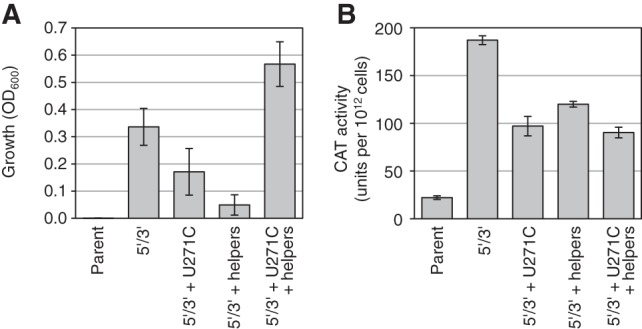
Activity and growth mediated by evolved spliceozyme mutations in *E. coli* cells. (*A*) Growth mediated by the spliceozyme variants in *E. coli* cells on medium containing 100 μg/mL chloramphenicol. Error bars are standard deviations from three experiments. (*B*) CAT enzyme activity of bacterial extract. The units correspond to 10^12^ cells. Error bars are standard deviations of three experiments. Note that spliceozyme W11 corresponds to the mutations 5′3′ + U271C + helpers.

To analyze the effect of the three groups of mutations on cellular function, two assays were performed (Fig. 4). Bacterial growth on plates containing a high concentration of chloramphenicol (100 μg/mL) increased strongly with the 5′- and 3′-terminal mutations from an undetectable level with the parent construct (Fig. 4A). While the further addition of the U271C mutation or the six helper mutations led to a decrease in growth, their combined addition led to maximum growth, showing that U271C acted cooperatively with at least a sub-set of the helper mutations. To test whether the improved growth of the evolved clone W11 on chloramphenicol containing medium was due to an increased production of the enzyme chloramphenicol acetyltransferase (CAT) the activity of this enzyme was measured in bacterial extract (Fig. 4B). Surprisingly, the U271C and helper mutations did not mediate an increase in CAT activity over the spliceozyme variant with only 5′- and 3′-terminal mutations. To reconcile the improved growth in the presence of chloramphenicol with reduced CAT activity, we hypothesized that in addition to facilitating CAT synthesis, spliceozyme activity may also impose a burden on the cell that reduces growth. The evolved U271C and helper mutations may have reduced this burden to mediate improved growth, possibly by a change in the pattern of product and side product formation, thereby reducing the production of and interference by nonfunctional RNA.

Two experiments were conducted to determine whether spliceozyme variants produced an effect on cellular growth independent of the antibiotic resistance mediated by splicing. First, bacterial growth in liquid culture was measured in the absence of chloramphenicol (Supplemental Fig. S2). Indeed, growth inhibition was observed for several spliceozyme variants when the *CAT* pre-mRNA was expressed together with the spliceozyme, compared with growth of cells expressing only these spliceozyme constructs without *CAT* pre-mRNA. Inhibition was observed when the spliceozyme variant was the parent, contained only the 5′ and 3′ mutations, or contained only the 5′, 3′, and helper mutations. No significant growth inhibition was observed if the spliceozyme contained the 5′, 3′, and U271C mutations, or if it contained the 5′, 3′, U271C, and helper mutations. Second, the splicing pattern of the parent and evolved W11 spliceozymes were compared when incubated with three essential *E. coli* mRNAs in vitro (Supplemental Fig. S3). Although no significant difference in the amount of cleavage products was detected, different cleavage patterns were observed. In summary, these results confirm that the spliceozymes can mediate an inhibitory effect on bacterial growth but it is currently unclear whether this effect is mediated by the cleavage of *CAT* pre-mRNA, off-target cleavage of endogenous *E. coli* mRNAs, or a different mechanism.

**FIGURE 5.**
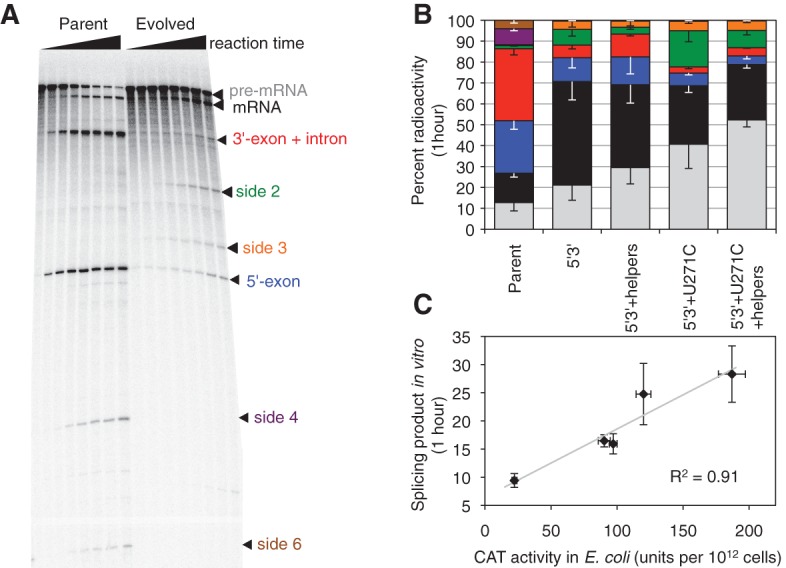
Effect of evolved spliceozyme mutations on the product pattern of in vitro splicing reactions. (*A*) Phosphorimage of radiolabeled splicing products with two different spliceozymes, separated by denaturing polyacrylamide gel electrophoresis. The *left* time course shows the products from the parent spliceozyme; the *right* time course shows the products from the evolved spliceozyme W11 (5′3′ + U271C + helpers). The time points are 0, 1, 2, 5, 10, 20, 30, and 60 min. The identity of the bands is given on the *right*. (*B*) Effect of evolved mutations in the spliceozyme on the product pattern of in vitro splicing reactions. The percentage of radioactivity in specific bands is plotted as a function of the spliceozyme construct, at a reaction time of 60 min. For a graph including all reaction times, see Supplemental Figure S4. Unreacted pre-mRNA (gray) is converted to mRNA (black). Cleavage products at the 5′-splice site are colored in blue (5′-exon) and red (3′-exon with intron). Additional side products are side2 (green), side3 (orange), side4 (purple), and side6 (brown). Error bars are standard deviations from three reactions. Note that the ribozyme variant labeled 5′3′ + U271C + helpers is identical to the evolved clone W11. (*C*) Correlation between product formation in vitro with CAT activity in *E. coli* cells. None of the other in vitro products was correlated similarly well with CAT activity or cell growth on chloramphenicol containing medium. Error bars are standard deviations from three experiments.

To test whether side product formation was reduced by the evolved spliceozyme, in vitro splicing reactions with radiolabeled substrate RNA were performed (Fig. 5). Splicing products were separated by denaturing polyacrylamide gel electrophoresis and the pattern of product bands quantified. One striking difference between the parent and evolved spliceozyme was the consumption of substrate; while the parent spliceozyme consumed ∼72% of substrate within 10 min of reaction time, the evolved spliceozyme W11 reacted only with 20% of substrate during the same time (Supplemental Fig. S4). Importantly, formation of the major two side products of the splicing reaction formed by cleavage at the 5′-splice site was significantly lower with the evolved spliceozyme (Fig. 5B). Four additional unidentified side products were observed during the in vitro reaction (side 2, 3, 4, and 6). Side products 2 and 3 were increased in the evolved spliceozyme W11 while bands 4 and 6 were decreased (Fig. 5B). In contrast, the helper mutations caused a significant reduction of side product 2 when added to the U271C mutation (compare 5′3′ + U271C with 5′3′ + U271C + helpers in Fig. 5B). This is consistent with the cooperative effect between U271C and the helper mutations observed in the growth of *E. coli* on chloramphenicol containing medium (Fig. 4A,B). Similarly, the amount of product formation in vitro correlated well with the amount of active CAT enzyme produced in *E. coli* cells (Fig. 5C). This suggested that the observed changes in splicing product patterns could be used to understand the phenotype in *E. coli*.

**FIGURE 6.**
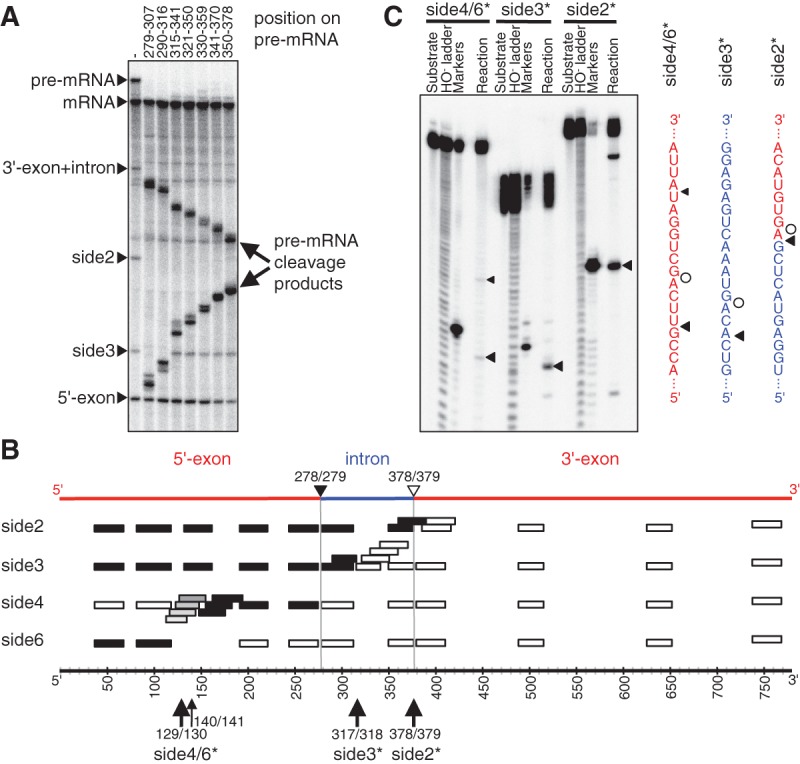
Identification of side products from the in vitro splicing reaction. (*A*) Phosphorimage of a denaturing PAGE separation of spliceozyme reaction products with internally ^32^P-labeled *CAT* pre-mRNA. After the reaction, the products were annealed with DNA oligonucleotides and treated with RNase H. The disappearance of a band showed that the DNA oligonucleotides had complementarity to the band's RNA. The positions covered by seven representative DNA oligonucleotides are shown above the gel image. These positions extend over the entire intron sequence. (*B*) Schematic of *CAT* pre-mRNA with the position of DNA oligonucleotides indicated. Effects of the DNA oligonucleotides on side product bands side2, side3, side4, and side6 are indicated by filled rectangles (band disappears) or empty rectangles (band is unaffected). Gray rectangles indicate partial disappearance. The position of 5′-exon (red), intron (blue), and 3′-exon (red) in the pre-mRNA are indicated on *top*. The nucleotide position is given on the *bottom*. The 5′-splice site is denoted by a black triangle; the 3′-splice site by an open triangle. Cleavage sites with single-nucleotide resolution (see (*C*)) are given on the *bottom*. (*C*) Identification of the cleavage sites at single-nucleotide resolution, using short substrate fragments with 5′-[^32^P] radiolabel. The sequences at which cleavage occurred are shown on the *right*, with triangles denoting cleavage sites by the spliceozyme and circles denoting the cleavage position by the DNAzyme to generate the marker. The resulting cleavage sites are annotated at the *bottom* of sub-figure (*B*).

To identify the RNA fragments that caused the observed changes in splicing product patterns in vitro (Fig. 5), an RNase H assay was performed (Fig. 6). RNase H digests only RNAs that form double strands with DNA (Davis et al. 1988). Products of the splicing reaction with radiolabeled substrate were annealed with DNA oligonucleotides designed to base pair with segments along the length of the *CAT* pre-mRNA and incubated with RNase H. When these digestion products were separated by denaturing PAGE alongside undigested splicing products (Fig. 6A), the bands that contained sequences complementary to the DNA oligonucleotides shifted down or disappeared. By employing more than a dozen DNA oligonucleotides, it was possible to identify the location of side products 2, 3, 4, and 6 along the length of the *CAT* pre-mRNA (Fig. 6B). Side product 2 consisted of the 5′-exon connected to the intron, therefore corresponding to the 5′-fragment of cleavage at the 3′-splice site. Side product 3 contained the 5′-exon and a portion of the intron, corresponding to the 5′-fragment of a cleavage event within the intron. Side products 4 and 6 corresponded to two complementary fragments of the 5′-exon.

The RNase H assay, however, did not allow for single-nucleotide resolution. To identify the cleavage sites with single-nucleotide resolution, truncated 5′-radiolabeled substrates were generated, incubated with the spliceozyme, and analyzed by denaturing PAGE (Fig. 6C). As marker, the same radiolabeled substrates were incubated with DNAzymes (Santoro and Joyce 1998) designed to cleave the substrates near the expected spliceozyme cleavage site. The results of this single-nucleotide resolution assay confirmed that side product 2 was cleaved precisely at the 3′-splice site, side product 3 had a 3′ terminus within the intron between nucleotides 317/318, and side products 4 and 6 were cleavage products of the 5′-exon between nucleotides 129/130, with perhaps a minor cleavage product at position 140/141 (see labels below Fig. 6B).

Together, these results suggested that the mutations at the spliceozyme 5′ and 3′ terminus, which extended the substrate recognition duplexes, reduced premature release of cleavage products at the 5′-splice site by 6.1-fold (after 1 h of splicing in vitro) and increased product (*CAT* mRNA) formation 3.5-fold (Fig. 5B). Cleavage products at the 5′-splice site were further reduced 2.0-fold by mutation U271C, which also increased cleavage at the 3′-splice site by 2.3-fold (side product 2). The six helper mutations reduced cleavage products at the 3′-splice site back by 2.1-fold. The overall effect of the evolved 5′ and 3′ terminus and U271C mutations was that of a rebalancing of activity between the 5′-splice site (reduction) and 3′-splice site (increase), with additional “helper” mutations to mitigate side effects from the increase in 3′-splice site activity.

## DISCUSSION

The activity of group I intron spliceozymes was improved by evolution in *E. coli* cells. Three types of mutations were identified that improve their performance in cells. First, mutations extending the substrate recognition helices (P1 and P9.2) increased product formation and decreased side product formation. Second, mutation U271C in the conserved core of the spliceozyme further reduced side products generated by cleavage at the 5′-splice site but increased side products generated by cleavage at the 3′-splice site. Third, a group of six “helper” mutations reduced side products formed by cleavage at the 3′-splice site. Together, the resulting spliceozymes show stronger product formation and weaker side product formation.

The effect of mutations extending the 5′-substrate recognition sequence can likely be explained by previous observations that extending the P1 helix can increase *trans*-splicing efficiency, possibly through an improved conformational change between the two catalytic steps (Kohler et al. 1999; Olson and Muller 2012). Similarly, it is expected that mutations at the 3′ terminus resulting in extension of the P9.2 helix lead to better retention of the splicing intermediates due to increased duplex stability.

**FIGURE 7.**
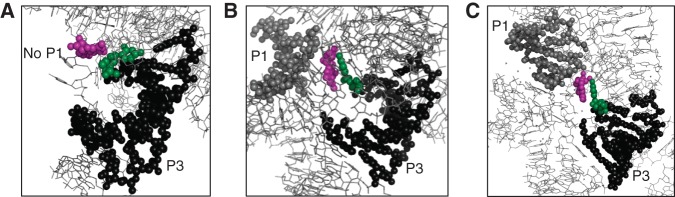
Crystallographic structures of group I introns that suggest a model for the function of mutation U271C. Shown are small portions of the structure that include the core helix P3 onto which U271 stacks. (*A*) The structure of a group I intron from *Tetrahymena* (1X8W) was made in the absence of the P1 helix. The nucleobase U271 (green spheres) is stacked onto helix P3 (black spheres) and points away from nucleotide G303 (purple spheres). The latter resides in the junction J7/8, which is known to assist the positioning of helix P1 into the catalytic site. (*B*) The structure of a group I intron from *Twort* (1Y0Q) was made in the presence of helix P1 (gray spheres). Here, the nucleotide analogous to U271 is stacked onto the nucleotide analogous to G303. (*C*) Similarly, the structure of a group I intron from *Azoarcus* (1ZZN) shows the stacking of the nucleobases analogous to U271 onto the base analogous to G303, in the presence of the P1 helix.

It is interesting that the U271C mutation, located in the conserved core of the group I intron, resulted in beneficial effects because mutations in the conserved core usually have detrimental effects of group I intron activity (Michel and Westhof 1990). The crystal structure of the *Tetrahymena* group I intron (Guo et al. 2004) provides a possible explanation as to how this mutation may have led to a decrease in activity of the 5′-splice site and an increase in activity of the 3′-splice site. This crystal structure shows nucleobase U271 stacked upon the end of the P3 helix, oriented towards A103 at the same end of the helix (Fig. 7A). This crystal structure, however, lacks the substrate recognition helix P1 and therefore the 5′-splice site. The P1 helix is present in the crystal structures of two related group I introns from the species *Azoarcus* and *Twort* (Fig 7B,C; Golden et al. 2005; Stahley and Strobel 2005). Here, the nucleobases analogous to U271 are flipped out of helix P3 and stack upon a G in the junction J7/8. The junction J7/8 is important for binding the substrate recognition helix P1 through ribose zipper interactions and positioning the 5′-splice site in the catalytic site (Silverman and Cech 1999). The P1 helix is known to flip in and out of the active site frequently (Shi et al. 2009), therefore influences on stabilizing the P1 helix to the catalytic site can be expected to have a strong influence on 5′-splice site activity. In summary, modulation of splice site activity by mutation U271C may have been caused by an indirect effect on positioning of the P1 substrate recognition helix into the catalytic site.

Two of the six “helper” mutations, A334G and G368Δ, are located at the base of the P9.2 helix at the entry point of the substrate intron into the catalytic site (Fig. 3B). It is possible that these two mutations serve to modulate the effect of increased activity at the 3′-splice site from the U271C mutation, thereby explaining the cooperative effect between U271C and the helper mutations (Fig. 4A). The mechanism of the four remaining helper mutations is less clear. These mutations reside in the periphery of the spliceozyme, and not close to the catalytic site, the substrate binding sites, or the U271C mutation suggesting that these four mutations may exert their effect via long-distance interactions. The evolved spliceozymes may therefore be a good model system to study how long-range interactions are able to modulate the function of catalytic RNAs.

It may be possible to develop spliceozymes that can treat certain types of mis-splicing disorders including intron and pseudoexon inclusion. In addition, certain disorders caused by mutations leading to premature termination or out of frame transcripts, could be treated by removal of non-essential portions of the mRNA; these too could be targeted by the spliceozyme. The evolved spliceozymes may be one step closer to possible applications in therapy because they now display an increase in product formation and decreased level of side product formation. The observation of these effects in vitro suggests that these results are not dependent on *E. coli-*specific cellular factors, and therefore may be applicable to a broad range of cell types. A safe and effective delivery method is still needed before this system could be applied clinically.

Evolution experiments of spliceozymes in cells can also be seen as a model system for the evolution of specific biochemical steps in the evolution of the spliceosome, which appears to have evolved from a common ancestor with group II intron ribozymes (Fica et al. 2013). While the “group I spliceozyme” model system is a long way from resembling the spliceosome's impressive ability to recognize with high precision and very low error rate thousands of different splice sites, the results shown here are encouraging that at least some of the biochemical steps in the evolution of the spliceosome could be recapitulated by the further evolution of these *trans*-splicing group I intron ribozyme variants.

## MATERIALS AND METHODS

### Library plasmid

Library plasmid was constructed essentially as described (Amini and Muller 2013) and expressed the indicated spliceozyme variant and the chloramphenicol acetyltransferase (*CAT*) pre-mRNA with a 100-nt intron inserted at position 258. Spliceozyme expression driven by the *trc2* promoter is inducible with IPTG, while the CAT pre-mRNA expression is driven by the constitutive promoter derived from the pLysS plasmid (Novagen). Both the spliceozyme and *CAT* pre-mRNA contain a 3′ hairpin terminator sequence.

### Evolution of spliceozymes in *E. coli* cells

The evolution was completed essentially as described (Olson and Muller 2012). Briefly, spliceozyme constructs were randomized by mutagenic PCR and cloned into library plasmid. Library plasmids containing spliceozyme sequences were transformed into *E. coli* cells, plated on LB agar medium containing 100 μg/mL ampicillin (amp) and incubated at 37°C for 16 h to create pools. LB agar plates containing pools of *E. coli* cells were washed using liquid LB medium. The resulting medium was diluted to an OD_600_ of 0.0015, induced with IPTG to a final concentration of 1 mM and shaken at 37°C for 1 h. *E. coli* cells were then plated on LB agar medium containing the indicated concentration of chloramphenicol and incubated at 37°C for 16 h to select for spliceozyme constructs able to mediate antibiotic resistance. LB agar plates were washed using liquid LB medium and plasmids were isolated by miniprep (5 Prime). Five or 10 clones were chosen for sequencing from each round of evolution.

### Generation of spliceozyme constructs by site-directed mutagenesis

Internal mutations were inserted into spliceozyme constructs by site-directed mutagenesis (Weiner and Costa 1994). Briefly, each 50 μL reaction contained 8 ng template plasmid, 1.25 pmol forward primer, 1.25 pmol reverse primer, 2.5 nmol each dNTP, 2.5 units PrimeSTAR GXL DNA Polymerse (Takara), and 1× PrimeSTAR GXL buffer. PCR consisted of 5′/95°C, 18 cycles of 50 min/95°C, 50 min/60°C, and 4.75 h/68°C, followed by 7 h/68°C. After PCR 20 units of DpnI restriction enzyme were added, the reaction was incubated at 37°C for 1 h and then purified (DNA clean and concentrate kit, Zymogen). Reactions were transformed into *E. coli* DH5α by electroporation.

### PCR mutagenesis

Mutations were randomly introduced into spliceozyme sequences using PCR mutagenesis (Cadwell and Joyce 1992). Each 100 μL reaction contained 25 ng template, 1 μM forward primer, 1 μM reverse primer, 10 mM Tris/HCl (pH 8.3), 50 mM KCl, 7 mM MgCl_2_, 0.05% gelatin, 0.2 mM dATP, 1.0 mM dCTP, 0.2 mM dGTP, 1.0 mM dTTP, 0.5 mM MnCl_2_, and Taq polymerase. PCR consisted of 30′′/94°C, and 10 cycles of 30′′/94°C, 30′′/50°C, 30′′/72°C, then 1′/72°C.

### Measurement of bacterial growth on LB agar plates

Measurement of bacterial growth on LB agar plates was completed essentially as described (Amini and Muller 2013). Five milliliters of liquid LB medium containing 100 μg/mL ampicillin was inoculated and grown at 37°C for 5 h. The OD_600_ of each culture was measured, diluted to an OD_600_ = 0.0025, induced with 1 mM IPTG and incubated, shaking at 37°C for 1 h. One hundred microliters of each culture was plated on an LB agar plate containing the indicated chloramphenicol concentration and an LB agar plate containing 100 μg/mL ampicillin. Plates were incubated at 37°C for 16 h then washed with 1.6 mL liquid LB medium. The OD_600_ of each wash was measured; measurements from ampicillin plates were used for normalization.

### Measurement of bacterial growth in liquid culture

Measurement of bacterial growth in liquid culture was completed essentially as described (Olson and Muller 2012). Overnight cultures of *E. coli* containing the library plasmid with the indicated spliceozyme variant were induced with 1 mM IPTG and incubated at 1 h shaking at 37°C. Each culture was diluted to an OD_600_ of 0.05 with LB medium containing 100 μg/mL ampicillin, induced with 1 mM IPTG and incubated, shaking at 37°C. OD_600_ measurements were taken after 1 h, and every following 30 min until an OD_600_ of 1.0 was reached. Doubling times were determined by least squares fitting of a single-exponential function.

### Assay for CAT activity from *E. coli* extracts

The measurement of CAT activity from *E. coli* extracts was completed essentially as described (Shaw 1975). Briefly, overnight cultures of *E. coli* containing the library plasmid with the indicated spliceozyme variant were induced with 1 mM IPTG and incubated 1 h shaking at 37°C. Each culture was diluted to an OD_600_ of 0.02 with LB medium containing 100 μg/mL ampicillin, and 1 mM IPTG and incubated, shaking at 37°C. When the OD_600_ reached 0.20, 2.0 mL of cells were concentrated to 200 μL and frozen. During the reaction, cells were thawed and mixed with 200 μL 200 mM Tris/HCl (pH 7.8), 10 mM Na_2_EDTA and 2 μL toluene. Fifteen microliters of this solution were diluted with 135 μL buffer to a final concentration of 0.2 mM chloramphenicol, 0.2 mM Acetyl-CoA and 1 mM DTNB. The A_412_ was then measured every 15 sec. The extinction coefficient of the reaction product (13,600 M^−1^ cm^−1^) was used to determine the units of CAT, with one unit of CAT being able to acetylate 1 μmol chloramphenicol/min.

### In vitro *trans*-splicing assays

In vitro *trans*-splicing assays were completed essentially as described (Amini et al. 2014). Spliceozyme and *CAT* pre-mRNA transcripts were generated by run-off transcription off PCR products and purified by denaturing PAGE. *CAT* pre-mRNA transcripts were internally labeled with [α-^32^P]-ATP. Twenty microliters splicing reactions contained 1 μM spliceozyme, 100 nM *CAT* pre-mRNA, 5 mM MgCl_2_, 50 mM MOPS/NaOH (pH 7.0), 2 mM spermidine, 135 mM KCl, and 20 μM GTP. Spliceozyme and *CAT* pre-mRNA were incubated separately for 10 min at 37°C then combined and incubated at 37°C. Two microliters samples were taken at 0, 1, 2, 5, 10, 20, 30, and 60 min and separated by 6% PAGE. Gel separations were imaged on a PMI phosphoimager (Bio-Rad) and bands were quantified using the software Quantity One. Signal strengths were normalized by the number of A's contained within each sequence and total radioactivity per lane. For the analysis of product pattern formation (Fig. 5B) only bands were considered that reached a level of 3% per lane for at least one of the constructs.

### Identification of in vitro splicing side products by RNase H (Fig. 6A)

In vitro *trans*-splicing reactions were run for 60 min (as described above) and quenched with EDTA to a final concentration 1.2-fold higher than MgCl_2_. Eight microliters of each reaction were added to 2 μL of the indicated DNA oligonucleotide, to a final concentration of 1 μM DNA. The oligonucleotide was allowed to anneal to the products of the splicing reaction by heating to 80°C for 2 min, followed by cooling to 25°C at 0.1°C/sec. Two μL MgCl_2_ were added to the reaction in stoichiometric equivalent to the EDTA present. Fifty microliters of RNase H reactions contained the 12 μL annealing reaction, 1× RNase H Reaction Buffer and 2.5 units RNase H (New England Biolabs) and were incubated at 37°C for 1 h. Reactions were purified by ethanol precipitation and separated by denaturing 6% PAGE. Gels were imaged on a phosphoimager (Bio-Rad). DNA oligonucleotides were designed to bind to the following nucleotide positions along the *CAT* pre-mRNA, where position 1 is the A of the translation start codon: 35–68, 81–108, 112–139, 117–144, 122–149, 127–154, 132–162, 147–177, 153–182, 158–190, 162–194, 191–221, 245–276, 279–307, 290–316, 315–341, 321–350, 330–359, 341–370, 350–378, 360–391, 370–401, 380–411, 385–416, 390–421, 487–516, 624–653, and 739–770.

### Identification of in vitro splicing side products with single-nucleotide resolution (Fig. 6C)

Each short substrate was incubated with an excess of spliceozyme to generate the cleavage products. Substrate RNA oligonucleotides (below) that contained the suspected splice sites as shown by the RNAse H assays, were generated by run-off transcription and purified by 10% PAGE. Substrates were dephosphorylated at 37°C for 1 h. Ten microliters of dephosphorylation reactions contained 2 ng substrate oligonucleotide, 1× Antarctic Phosphatase Reaction Buffer and 5 units Antarctic Phosphatase (New England Biolabs). Substrates were then 5′ radiolabeled at 37°C for 1 h. Twenty microliters labeling reactions consisted of 20 pmol RNA substrate, 20 μCi [γ-^32^P]-ATP, 1× T4 Polynucleotide Kinase Reaction Buffer and 10 units T4 Polynucleotide Kinase (New England Biolabs). Radiolabeled substrates were purified by denaturing PAGE. DNAzymes (below) were designed to cleave substrate oligonucleotides between the underlined AG position to create markers. Twenty microliters of DNAzyme reactions consisted of traces of labeled substrate oligonucleotides, 1 μM DNAzyme, 150 mM NaCl, 100 mM MgCl_2_, and 50 mM Tris/HCl (pH 8.3). Reactions were heated to 80°C for 2 min, then incubated at 37°C for 1 h. MgCl_2_ and Tris/HCl were added after reactions reached 37°C. Reactions were separated by denaturing 6% PAGE and gels were imaged on a phosphoimager (Bio-Rad). Note that hydroxyl ladders and the marker created by a DNAzyme carry 3′-phosphates. This difference to the 3′-hydroxyl groups created by the spliceozyme makes the spliceozyme cleavage products migrate slower, corresponding to ∼0.4 nt. The following oligonucleotide and DNAzyme sequences were used:

Side 2 oligonucleotide: GGUGUAAGCUCUCCCCUUGCAGAUUAGUUUUGGAGUACUCGAGUGUACACCCUUGUUACACCGUUUUCCAUGAGCAAACUGAAA
Side 2 DNAzyme: AACAAGGGTGTACACTCCGAGCCGGACGAGAGTACTC
Side 3 oligonucleotide: GAAGGGUUCCUGGGAAGGCCUUCGGGUCACAGUAAACUGAGAGGCUUUGGUG
Side 3 DNAzyme: CACCAAAGCCTCTCCGAGCCGGACGAAGTTTACT
Side 4 and side 6 oligonucleotide: AGUUGCUCAAUGUACCUAUAACCAGACCGUUCAGCUGGAUAUUACGGCCUUUUUAAAGACCGUAAAGAAAAAUAAGC
Side 4 and 6 DNAzyme: AAAAAGGCCGTAATATCCGAGCCGGACGACAGCTGAA

### In vitro splicing reactions on three essential *E. coli* mRNAs (Supplemental Fig. S3)

*E. coli* genomic DNA was prepared by proteinase K digestion of the cell pellet from a logarithmically grown culture of *E. coli* DH5α, and two consecutive ethanol precipitations. The desired gene fragments were amplified from this preparation of genomic DNA by PCR. Transcripts of the first 602, 586, and 633 nt of *E. coli* EF-Tu, Pyruvate Dehydrogenase Subunit E2, and DNA Polymerase III Subunit α genes, respectively, were generated by run-off transcription from PCR products and purified by PAGE. The nucleotides GGGAG were added to the 5′ end of each substrate to enhance transcription efficiency. Transcripts were internally labeled by transcription in the presence of [α-^32^P]-ATP. Splicing reactions with a volume of 20 μL contained 1 μM spliceozyme, 100 nM of the indicated RNA substrate, 5 mM MgCl_2_, 50 mM MOPS/NaOH (pH 7.0), 2 mM spermidine, 135 mM KCl, and 20 μM GTP. Spliceozyme and RNA substrate were incubated separately for 10 min at 37°C then combined and incubated at 37°C. Samples with a volume of 2 μL were taken at 0, 1, 2, 5, 10, 20, 30, and 60 min and separated by denaturing 6% PAGE. Gels were imaged on a phosphoimager (Bio-Rad) and bands were quantified (Quantity One). Signal strengths were normalized by the total radioactivity per lane.

## SUPPLEMENTAL MATERIAL

Supplemental material is available for this article.

## Supplementary Material

Supplemental Material
