# Supplementary material for: Increased efficiency of evolved group I intron spliceozymes by decreased side product formation
Source: RNA. 2015 Aug;21(8):1480–9. doi: 10.1261/rna.051888.115 (PMC4509937; doi:10.1261/rna.051888.115)
Supplement: Supplemental Material [file supp_21_8_1480__index.html]

Increased efficiency of evolved group I intron spliceozymes by decreased side product formation — Increased efficiency of evolved group I intron spliceozymes by decreased side product formation — Supplemental Material 

# Increased efficiency of evolved group I intron spliceozymes by decreased side product formation

## Supplemental Material

**Files in this Data Supplement:**

- Supp Figures S1-S4.pdf
