# Supplementary material for: Increased efficiency of evolved group I intron spliceozymes by decreased side product formation
Source: RNA. 2015 Aug;21(8):1480–9. doi: 10.1261/rna.051888.115 (PMC4509937; doi:10.1261/rna.051888.115)
Supplement: Supplemental Material [file supp_051888.115_SuppFigsS1-S4.pdf]

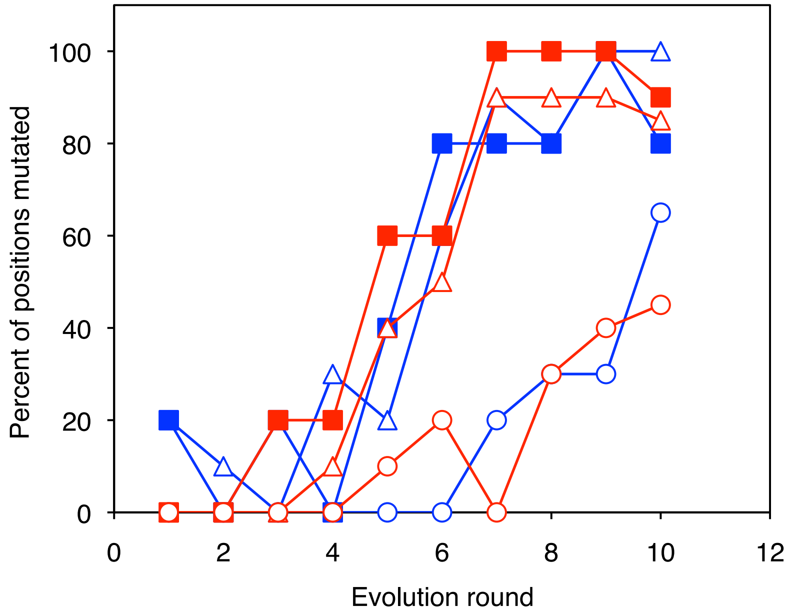

Figure S1. Enrichment of key mutations during evolution. Shown is the percentage of spliceozyme clones that were mutated at a given position. Data from the line starting at a single sequence are in blue; data from the pre-evolved line are in red. Mutations at the two 5'-terminal positions are labeled as open triangles, the U271C mutation is labeled with filled squares, and mutations at the two 3'-terminal positions are shown as open circles. In rounds 1-9 of the evolution, five clones were sequenced; in round 10, ten clones were sequenced.

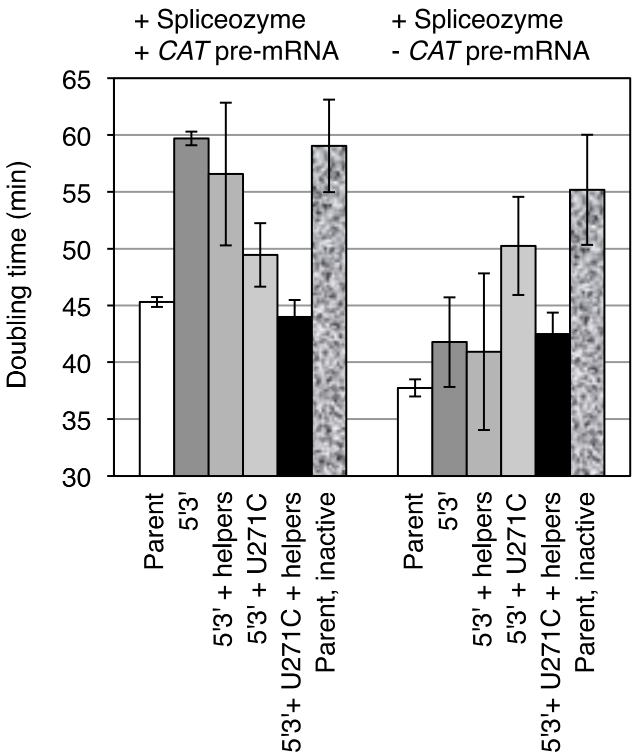

Figure S2. Growth inhibition of *E. coli* via the expression of spliceozyme variants and CAT pre-mRNA. Growth was measured by the increase in OD_{600} in liquid medium in the absence of chloramphenicol. Six spliceozyme variants were tested, in the presence (left) or absence (right) of CAT pre-mRNA. The spliceozyme variants are labeled below the graph. Error bars are standard deviations from biological triplicates.

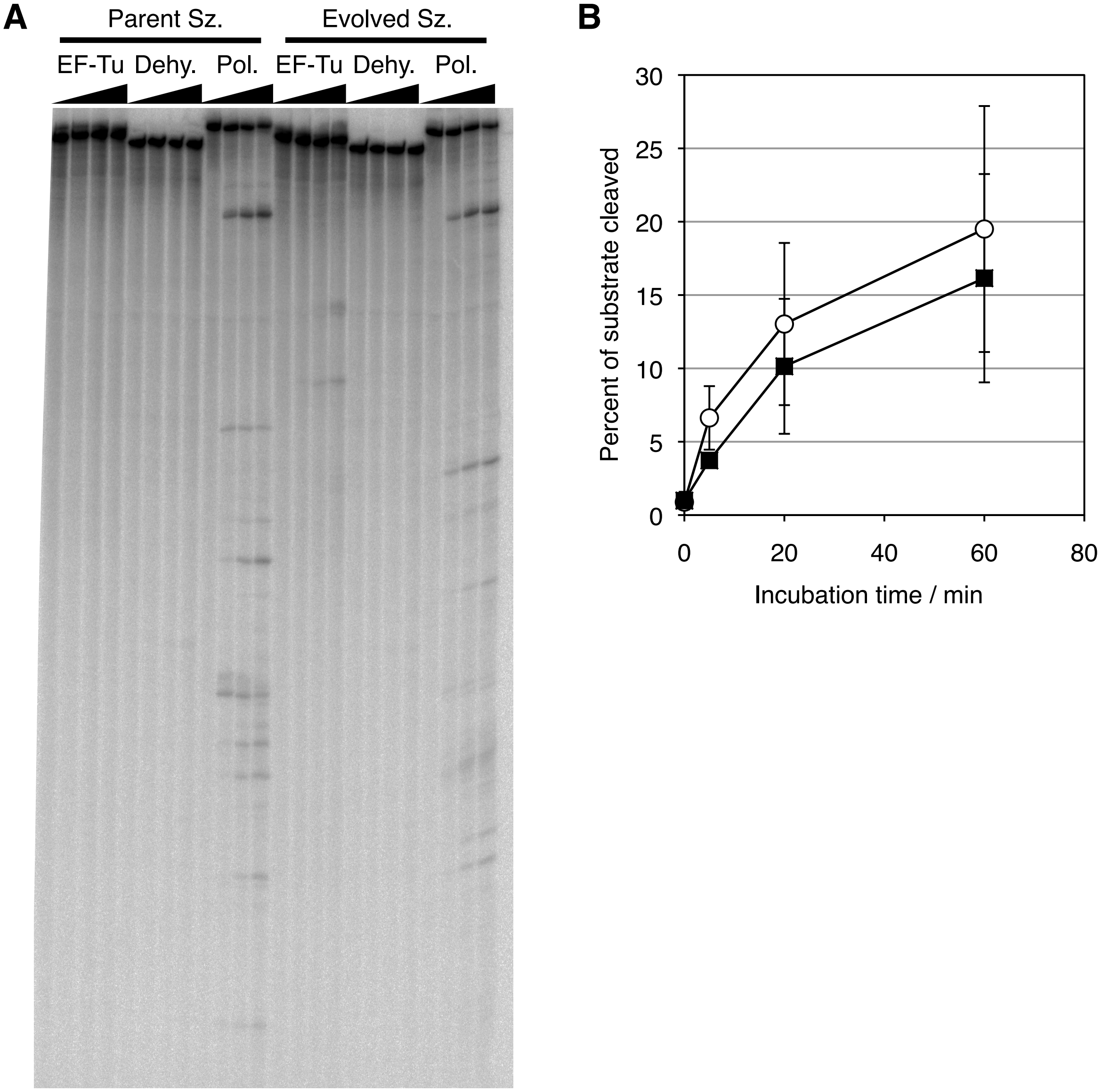

Figure S3: Off-target effects of the spliceozyme on three essential mRNAs in *E. coli*. **(A)** Phosphorimage of internally radiolabeled RNAs after incubation with spliceozyme. The sequences are from the mRNAs of E. coli elongation factor Tu (EF-Tu), *E. coli* pyruvate dehydrogenase sub-complex E2p (Dehy.), and *E. coli* DNA polymerase 3 (Pol.). The black triangles illustrate increasing incubation times (0 min., 5 min., 20 min., and 60 min.) with parent spliceozyme (Sz) or evolved spliceozyme. **(B)** Quantitation of the cleavage activity of DNA polymerase mRNA. The intensity of all bands visible for the parent spliceozyme (empty circles) and the evolved spliceozyme (black squares) is plotted as a function of incubation time with the spliceozyme. Error bars are standard deviations from three experiments.

Figure S4

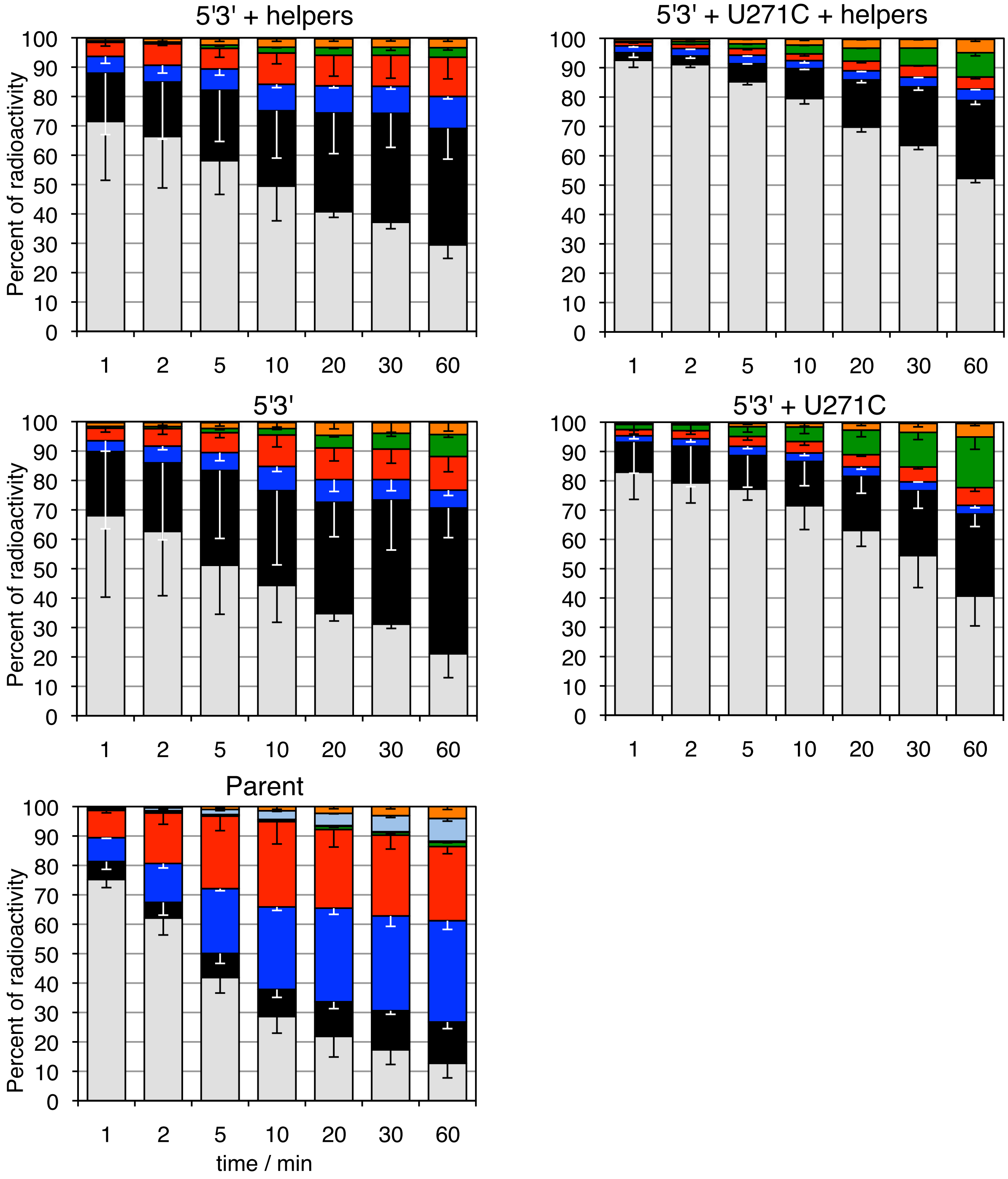

Figure S4: Effect of evolved spliceozyme mutations on the product pattern of *in vitro* splicing reactions. The percentage of radioactivity in specific bands is plotted as a function of the spliceozyme construct, at at time points of 0, 1, 2, 5, 10, 20, 30, and 60 minutes. Unreacted pre-mRNA (grey) is converted to mRNA (black). Cleavage products at the 5'-splice site are colored in blue (5'-exon) and red (3'-exon with intron). Additional side products are side2 (green), side3 (orange), side4 (purple), and side6 (brown). Error bars are standard deviations from three reactions.
